# Interventions to improve resilience in physicians who have completed training: A systematic review

**DOI:** 10.1371/journal.pone.0210512

**Published:** 2019-01-17

**Authors:** Carolina Lavin Venegas, Miriam N. Nkangu, Melissa C. Duffy, Dean A. Fergusson, Edward G. Spilg

**Affiliations:** 1 School of Epidemiology, University of Ottawa, Ottawa, Ontario, Canada; 2 Department of Educational Studies, University of South Carolina, Columbia, South Carolina, United States of America; 3 Ottawa Hospital Research Institute, Ottawa, Ontario, Canada; 4 Department of Medicine, University of Ottawa and The Ottawa Hospital, Ottawa, Ontario, Canada; Norwegian University of Science and Technology, NORWAY

## Abstract

**Background:**

Resilience is a contextual phenomenon where a complex and dynamic interplay exists between individual, environmental, and socio-cultural factors. With growing interest in enhancing resilience in physicians, given their high risk for experiencing prolonged or intense stress, effective strategies are necessary to improve resilience and reduce negative outcomes including burnout. The objective of this review was to identify effective interventions to improve resilience in physicians who have completed training, working in any setting.

**Methods and findings:**

We included randomized controlled trials (RCT), and observational studies (including pilot studies) published in English, French, and Spanish that included an intervention to improve resilience in physicians who have completed training. We included studies that implemented interventions to reduce burnout, anxiety, and depression or to improve empathy to ultimately enhance resilience, rather than studies designed solely to reduce stress or trauma-induced stress. We performed a systematic search of Medline, EMBASE, PsychInfo, CINAHL and Cochrane Library with no publication year limit. The last search was conducted on March 29, 2017. We used random effect models to calculate pooled standardized mean differences. Resilience was the primary outcome measure using validated resilience scores. Secondary outcome measures included proxy measures of resilience such as burnout, empathy, anxiety and depression. Our search strategy identified 7,579 records;74 met the criteria for full-text review. Seventeen studies were included in the final review published between 1998 and 2016 of which 9 (4 RCT, 5 observational) had physician data extractable. Interventions varied greatly regarding their approach, duration, and follow-up. Two RCTs measured resilience using validated scales; both found a significant improvement. No meta-analysis for resilience was conducted due to the presence of high clinical and methodological heterogeneity.

**Conclusions:**

Our systematic review demonstrates that there is weak evidence to support one intervention over another to improve resilience in physicians who have completed training. The quality of evidence for the outcomes ranged from very low to low. There is a need for a consensus on the definition of resilience and how it is measured. Longer follow-up is required to ensure any intervention effects are sustained over time.

## Introduction

Resilience refers to the act of coping, adapting, or thriving from adverse or challenging events, where a complex and dynamic interplay exists between individual, environmental and socio-cultural factors. Thus interventions towards improving resilience should be geared towards, individual, group and organizational levels [1–4]. It negatively relates to various psychological morbidities ranging from burnout to depression, and frequently overlaps with the concept of wellness [1]. To assess the effectiveness of various resilience interventions, one must understand the factors that impact resilience, as well as the definitions of this construct.

Personal factors including personality, previous experience of adversity, coping strategies, and organizational factors, including workload and hours, play an important role in predicting resilience [5]. However, the culture of the more immediate social network within which an individual operates is a third factor, with the stigma associated with a doctor suffering psychological problems reducing resilience [1].

Several definitions for the concept of resilience are described with a wide range of scales used for measuring resilience [6–12]. In this review, we focus on resilience as a continuous, effective and positive adaptation process to adversity, rather than the absence of burnout alone [9].

Enhancing resilience in stressful occupational settings is of growing interest given that these individuals may be more likely to experience adversity increasing burnout risk. Physicians, in particular, are at higher risk of stress [13]. Effective strategies are necessary to prevent negative outcomes including anxiety, depression, burnout, relationship problems, suicide ideation and alcohol abuse [9,14,15]. There are also negative organizational outcomes including impaired work performance and high turnover [16]. Resilience has been identified as an important factor that could help to prevent these consequences and also potentially improve patient clinical outcomes [17–19].

With respect to previous reviews of resilience research a recent systematic review on resilience only studied primary healthcare professionals and publications were limited from 1994 to 2014 with a focus on definitions, measures and associations, rather than interventions [9]. One systematic review and meta-analysis on the efficacy of resilience training programs studied randomized trials on diverse adult populations (not specifically healthcare) and persons with chronic diseases [20]. Another systematic review on educational interventions to improve resilience focused on educational programmes, for healthcare students and professionals [21]. Finally, a systematic review of interventions to foster physician resilience among physicians included those still in training [22]. In contrast to these reviews, we focus specifically on resilience-building interventions to enhance resilience in physicians who have completed training, working in all settings. Although resilience should be enhanced at all stages of the medical career, we have excluded residents and medical students in this review as they represent distinct groups from physicians in independent practice. As they operate within educational and training environments, it is likely that interventions may impact these groups differently. We conducted a systematic review and meta-analysis of published studies of interventions to improve resilience in physicians who have completed training, working in any setting.

## Methods

### Search strategy and study eligibility

The protocol for this systematic review and meta-analysis was registered in PROSPERO (CRD42017060197). The conduct of this systematic review and meta-analysis is reported in adherence to the guidelines for systematic review and meta-analyses using the preferred reporting items for systematic review and meta-analyses (S1 **Checklist**) [23,24]. We included randomized controlled trials (RCTs) and observational studies published in English, French and Spanish that included an intervention to improve resilience in physicians who have completed training working in any setting. We were primarily interested in studies that implemented interventions to enhance resilience and which presented outcome data related to resilience and not just with the intention of reducing stress. Studies with the aim of enhancing resilience but which did not specifically measure resilience i.e. which measured a ‘proxy’ for resilience such as burnout, depression, anxiety and empathy were included as our secondary outcome measures as these studies targeted these states with the aim of enhancing resilience without specifically measuring resilience. While it is sometimes assumed that burnout and resilience are at opposite ends of the same spectrum, they are separate distinct entities with different measurement scales rating different concepts. Other studies which focused on stress reduction alone (unrelated to resilience) were excluded i.e. when it was clear that they were measuring more short-term or specific interventions which did not really apply to resilience (e.g. when they were interventions to reduce anxiety/stress for breaking bad news specifically, or for distress following patient adverse events, or when they measured states such as phobic anxiety) because stress and burnout are two separate but intertwined entities. Stress does not necessarily cause burnout, but it is not possible to be burned out without experiencing stress [25]. Studies that only included interventions to improve resilience in residents and medical students were ineligible. However, any study that included physicians in addition to residents, medical students or other health care providers was eligible for inclusion, but the extraction of data depended on whether there was subgroup analysis for physicians. For the outcome, we included studies that measured resilience based on the definitions above and the outcome measures as stated in our PICO. Commentaries, perspectives, expert opinions, conference proceedings, editorials, book chapters, and theses were all excluded.

Medline, EMBASE, PsychInfo, CINAHL and Cochrane Library were searched with the help of an information specialist trained in conducting systematic reviews (S1 Database). There was no publication year limit, and the last search was conducted on March 29^th^, 2017. We also searched in Google Scholar, BMJ Careers and grey literature. Clinical trial registries were searched to identify completed and in-progress studies. We contacted study authors and hand-searched relevant study references. The full electronic search strategy for all databases is available in S1 Database.

Screening, eligibility and inclusion assessment was conducted independently by two reviewers (CL, MN). Search results were exported into Endnote for duplication removal and referencing and then exported to Covidence for independent screening and additional removal of duplicates. Initial screening involved assessment of abstracts and titles only. Full eligibility was then assessed through full-text screening. Any disagreements were resolved by consensus. When consensus was not achieved, additional reviewers (ES, DF) were consulted. In cases where a study had multiple publications, the most recent one was retained.

### Data extraction and quality assessment

Data from included publications was extracted independently by the two reviewers (CL, MN) using a data extraction form piloted. Any disagreement was resolved by consensus, and when consensus was not achieved, the other reviewers (ES, DF) were consulted. Authors of the included studies were contacted when extraction was unclear or when data was missing. Specific data extracted included the study author, publication year, study design, setting, and sample size (number of participants invited, enrolled, randomized and analyzed according to study design). Demographics included the population studied such as physicians’ level of care/specialty (primary, secondary or tertiary), mean age and gender. We described the type and details of the interventions and comparators such as duration and frequency. We included outcome measures with their corresponding scales, along with measurement time points and follow-up.

We also extracted data regarding missing data, funding sources and risk of bias. We used the Cochrane Collaboration risk of bias tool for RCTs [26], and the Cochrane Risk of Bias Assessment Tool for Non- Randomized Studies of Interventions (ACROBAT-NRSI) [27] for non-randomized studies. We extracted post-intervention means and standard deviations (SD) for RCTs. For observational studies, we extracted pre-intervention and post-intervention means and SDs. When SDs were not reported, we calculated SDs from confidence intervals (CI) if provided. For outcomes that were reported using different scales, we converted the difference in means (MD) to standardized mean differences (SMD).

### Data aynthesis and analysis

All data was analyzed using RevMan 5.3. Before pooling results, we assessed clinical and methodological heterogeneity. Due to extensive heterogeneity, pooling of data was limited to resilience and burnout outcome measures. RevMan 5.3 was used to conduct meta-analyses using random effects and to calculate the I^2^ according to the Cochrane Handbook thresholds, where >50% was considered substantial heterogeneity [26]. Publication bias using funnel plots was not assessed due to insufficient number of studies. Instead, we critically appraised the evidence, focusing on broadness of the search, selective outcome reporting and other sources of bias. To evaluate the quality of evidence for our outcome measures, we used the grade recommendation assessment development and evaluation (GRADE) approach, separately for RCTs and observational studies [28]. We evaluated the quality of evidence using the risk of bias, inconsistency, indirectness, imprecision and publication bias. We reported the quality as very low, low, moderate or high. We conducted pre-specified subgroup analyses separating studies that reported resilience measured by validated resilience scores from studies reporting secondary outcome measures of resilience. We also conducted planned subgroup analysis based on level of care (primary, secondary or tertiary) and study design.

## Results

### Study characteristics

**Fig 1:**
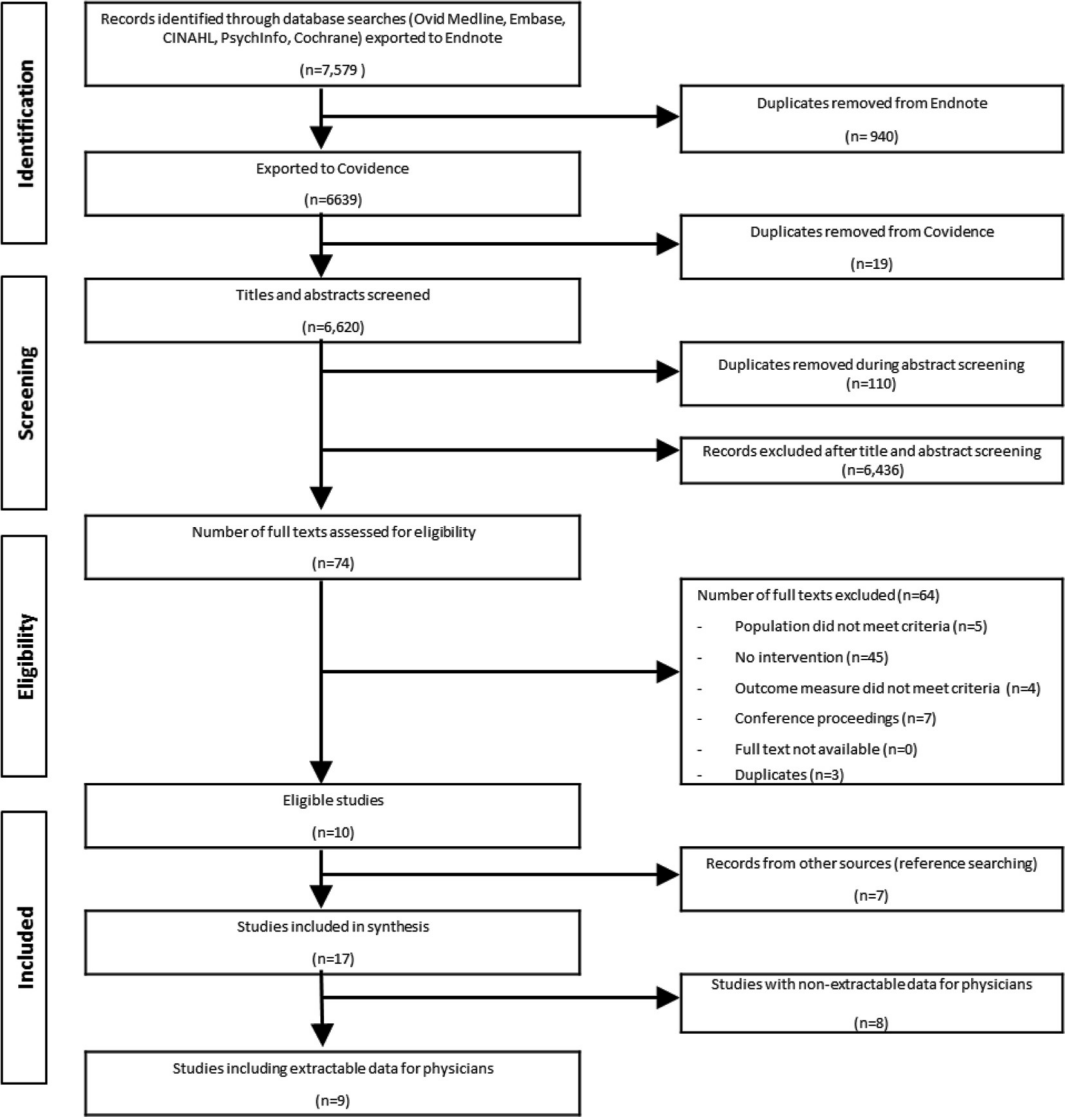
Prisma flow diagram.

We identified 7,579 records in our search strategy; 74 met the criteria for full-text review. The PRISMA flow diagram is presented in Fig 1. Seventeen studies were included in the final review. Eight studies [26–36] included other health care providers or residents, but did not provide subgroup analysis for physicians, and thus their results were not analyzed (S2 Table). Ten authors of the included studies were contacted (twice if necessary) due to unclear or missing data results, or no physician subgroup analysis. One author provided physician subgroup data. For the others that replied, no subgroup analysis was provided. Thus, 9 eligible physician-specific studies [15,37–44] were analyzed.

A summary of the clinical characteristics for the 9 eligible studies is presented in Tables 1 and 2. A summary of the methodological characteristics is presented in S1 Table. In general, the studies were published between 1998 and 2016. Four were randomized controlled trials (RCT) [15,37,43,44], 2 of which were pilot RCTs [15,37], one of which [15] was a wait-list controlled trial. Five articles [38–42] were observational studies, one of which [42] could not be analyzed as we were unable to extract the data, as the results were only graphical presentations of the outcome measures and p-values. These five were before-and-after studies with no control groups, except for one [42]. Five studies were in the United States [15,39,40,43,44], 3 of which [15,43,44] were conducted by the Department of Medicine at the Mayo Clinic. Three studies [37,41,42] were conducted in Europe (Norway, UK, and Germany). One study [38] was conducted in Australia.

**Table 1:** Summary of clinical characteristics of studies including results specifically for physicians in randomized controlled trials.

Author/Year/Design	Population/Setting	Mean age and gender	Outcome measures/ scales	Intervention/ description	Relevant findings associated with resilience
Dyrbye et al. 2016[43] (RCT)	Physicians from the Mayo Clinic Departments of Medicine in Minnesota and Arizona and from the Mayo Clinic Department of Surgery in Minnesota, USA.	Minimum age was 31 years IG 64.4% males CG 70.8% males	Burnout (Maslach Burnout Inventory)^a^, depression (2-item Primary Care Evaluation of Mental Disorders), meaning in work (12-item Empowerment at Work Scale), work engagement (six-item absorption sub-scale of the Work Engagement Scale), quality of life (single-item linear analog scale assessment), job satisfaction (Physician Job Satisfaction Scale), and fatigue (one-item standardized linear analogue scale)	Online, self-directed micro-tasks specifically crafted for physicians and intentionally designed to cultivate professional satisfaction and well-being in 6 domains. Physicians were asked to select and complete one task weekly. Once a week for 10 weeks.	No statistically significant decrease in burnout or depression
Mache et al. 2016[37] (RCT)	Psychiatrists from 12 hospital departments in north Germany	33 years IG 28% males CG 31% males	Resilience (Brief Resilient Coping Scale), Job satisfaction (Copenhagen Psychosocial Questionnaire), perceived stress (Perceived Stress Questionnaire), and self-efficacy (Questionnaire of Self-Efficacy, Optimism and Pessimism)	Psychosocial skills training combined with cognitive behavioural and solution-focused counselling 12 weekly sessions of 1.5 hours, performed off duty	Significant improvements in resilience at both follow-up surveys with no comparable results seen in the control group
Sood et al. 2011^b^ [15] (RCT)	Tertiary care physicians from the Department of Medicine at the Mayo Clinic in Rochester, USA	IG 46.8 years 55% males CG 50.2 years 50% males	Resilience (Connor Davidson Resilience Scale), perceived stress (Perceived Stress Scale), anxiety (Smith Anxiety Scale) and overall quality of life and fatigue (Linear Analog Self Assessment Scale)	Stress Management and Resiliency Training (SMART) program adapted from the Attention and Interpretation Therapy (AIT) structured therapy developed at Mayo Clinic, in addition to a brief structured relaxation intervention (pace breathing meditation One 90 min session and an optional 30–60 min extra follow up session	Statistically significant improvement for resilience and anxiety in the study arm compared to the wait-list control arm
West et al. 2014[44] (RCT)	General internal medicine and other internal medicine specialty physicians in the Department of Medicine at the Mayo Clinic in Rochester, USA	No mean age reported IG 32.4% female CG 35.1% female	Burnout (Maslach Burnout Inventory), depression (2-question approach described by Spitzer et al and validated by Whooley et al.), empathy (Jefferson Scale of Physician Empathy), meaning in work, empowerment and engagement in work (Empowerment at Work Scale), quality of life (single-item linear analog scale assessment), job satisfaction (Physician Job Satisfaction Scale), and perceived stress (Perceived Stress Scale)	Facilitated physician discussion groups incorporating elements of mindfulness, reflection, shared experience, and small-group learning 1-hour meetings occurring once every 2 weeks for 9 months, for a total of 19 sessions	No statistically significant differences in empathy, and depression For burnout, rates of emotional exhaustion and overall burnout were small, but the rate of high depersonalization 3 months following the study had a statistically significant decrease in the intervention arm compared to the control group. The difference was sustained at 12 months

Abbreviations: IG, intervention group; CG, control group.

Note: No studies had active comparators and all results reported for change from baseline to end of study.

^a^ Maslach Burnout Inventory subscales: emotional exhaustion, depersonalization and personal accomplishment.

^b^ Study had a wait-list control.

**Table 2:** Summary of clinical characteristics of studies including results specifically for physicians in observational studies.

Author/Year	Population/Setting	Mean age and gender	Outcome measures/ scales	Intervention/ description	Relevant outcomes results associated with resilience
Goodman et al. 2012[39]	Physicians and other healthcare providers from Charlottesville, Virginia and Rochester, USA, representing 11 different specialties including primary care physicians	Unclear mean age and gender for practicing physicians specifically	Burnout (Maslach Burnout Inventory), and self-perceived physical and mental health (SF-12v2)	Mindfulness Based Stress Reduction (MBSR) for healthcare providers 2.5 hours a week for 8 weeks, and included a 7-hour silent retreat between the 6th and 7th weeks	Burnout scores improved significantly from the first to the last class for physicians
Krasner et al. 2009[40]	Primary care physicians in the Greater Rochester, New York, USA	No mean age reported. 54% Males	Burnout (Maslach Burnout Inventory), empathy (Jefferson scale of physician), mindfulness (2-Factor Mindfulness Scale), psychosocial orientation (Physician Belief Scale), personality and mood (Mini-markers of the Big Five Factor Structure)	Intensive educational program in mindfulness, communication, and self-awareness An 8-week intensive phase (2.5 hour/week, 7-hour retreat) was followed by a 10-month maintenance phase (2.5 hour/month)	Burnout showed improvement across all subscales Total empathy improved
Isaksson et al. 2010[41]	Physicians who attended a counselling intervention for burnout at the Resource Center Villa Sana in Norway (primary and secondary care physicians)	46.8 years 45% males	Level of emotional exhaustion (5-point subscale emotional exhaustion of Maslach Burnout Inventory), perceived job stress (modified version of the Cooper Job Stress Questionnaire), coping strategies (Vitaliano and colleagues' Ways of Coping) and personality (Eysenck's abbreviated personality questionnaire)	Two types of interventions based on an integrative approach incorporating psychodynamic, cognitive, and educational theories 1) Single day, 6 to 7-hour counselling session for one physician with a psychiatrist or a specialist in occupational medicine 2) Five day, group based course for 8 participants, led by one of the same counsellors in collaboration with an occupational therapist	There were significant changes in levels of emotional exhaustion (Burnout subscale) from baseline to one year after the intervention, and were maintained at 3-year follow-up
Sherlock et al. 2016[42]	General practitioners (primary care) in the UK who had scores ≥ 8 on the Hospital Anxiety and Depression Scale (HADS) at baseline	No mean age reported	Anxiety and depression (HADS) and stress (Simple Stress Scale)	Course on adaptation practice which is a behavioural programme of self-discipline designed to cope with stress, anxiety and depression 6-month training course	HADS scores for anxiety and depression improved significantly compared with those of the control group
Winefield et al. 1998[38]	Female general practitioners (primary care) in Australia	39.6 years 100% females	Burnout (Maslach Burnout Inventory), level of psychological distress (12-item form of the General Health Questionnaire), and job satisfaction (modified form of the 16-item questionnaire by Wan et al., 1979).	Three, 3-hour meetings in 4 weeks	Significant reduction of emotional exhaustion (burnout subscale)

Note: No studies had active comparators and all results reported for change from baseline to end of study.

The physician population included physicians working across all settings and various specialties. Four [39,40,42,44] did not report mean age, and for those that did, age ranged between 31 and 50 years. Two [39,42] did not report gender specifically for physicians; one study [37] had substantially more females, and two studies [43,44] had substantially more males. One [38] only included females. The sample sizes ranged from 40 to 290 participants.

### Resilience-building interventions

Interventions for building resilience varied across studies. One RCT undertook a psychosocial skills training and cognitive behavioural approach [37]. Another RCT studied the Stress Management and Resiliency Training (SMART) program, developed at the Mayo Clinic to decrease stress and enhance resilience [15]. One trial facilitated physician discussion groups, mindfulness, reflection and small group learning [44]. Another trial provided an online self-directed micro-tasks intervention specifically for physicians [43]. One observational study conducted an intensive educational program in mindfulness and communication [40]. A prospective cohort study offered two intervention options, both of which were based on an integrative approach incorporating psychodynamic, cognitive and educational theories [41]. Another observational study involved a course in adaptation practice to learn how to cope with stress, anxiety, and depression [42]. Another study offered a paid Mindfulness Based Stress Reduction (MBSR) course for healthcare providers [39]. The intensity of interventions also varied, ranging from a single 90-minute session, to repeated sessions across several months. Follow-up ranged from a minimum of 8 weeks, to a maximum of 3 years. Three studies [40,43,44] offered monetary incentives, while another [40] offered Continuing Medical Education credits.

Two studies reported our primary outcome, resilience; one used the Brief Resilient Coping Scale [37] and the other the Connor-Davidson Resilience Scale [15]. Both are validated resilience scales. Seven reported secondary outcome measures [15,39–44] (burnout, anxiety, empathy and/or depression) but not specifically resilience. Burnout was measured in four studies [40,41,43,44] with the Maslach Burnout Inventory (MBI), which has three subscales, emotional exhaustion (EE), depersonalization (DP) and personal accomplishment (PA). However, one of these only measured the emotional exhaustion subscale [41].

Two pilot RCTs [15,37] reported our primary outcome measure of resilience measured by validated resilience scales (Tables 1 and 2). Both pilot trials [15,37] reported significant improvement in resilience. Six studies [38–41,43,44] reported burnout scores using the 3 subscales of the MBI, except Isaksson who used the emotional exhaustion 5-item subscale of the MBI. Of these 6 studies, the two RCTs [43,44] reported no statistically significant differences in the three subscales of burnout, except DP which was reported to be significant in one of the studies [44] but for which data could not be extracted for meta-analysis. The 4 observational studies [38–41] reported significant improvements in the EE subscale for burnout. Two studies [39,40] reported significant improvements for the DP and PA burnout subscales. No statistical significance was reported for depression in the two RCTs [43,44]. One observational study [42] reported significant improvements in depression and anxiety. One RCT measured anxiety, reporting a statistically significant improvement [15]. Two studies measured empathy, where the RCT [44] reported no statistically significant improvement, while the observational study [40] reported significant changes.

### Synthesis of results

**Fig 2:**
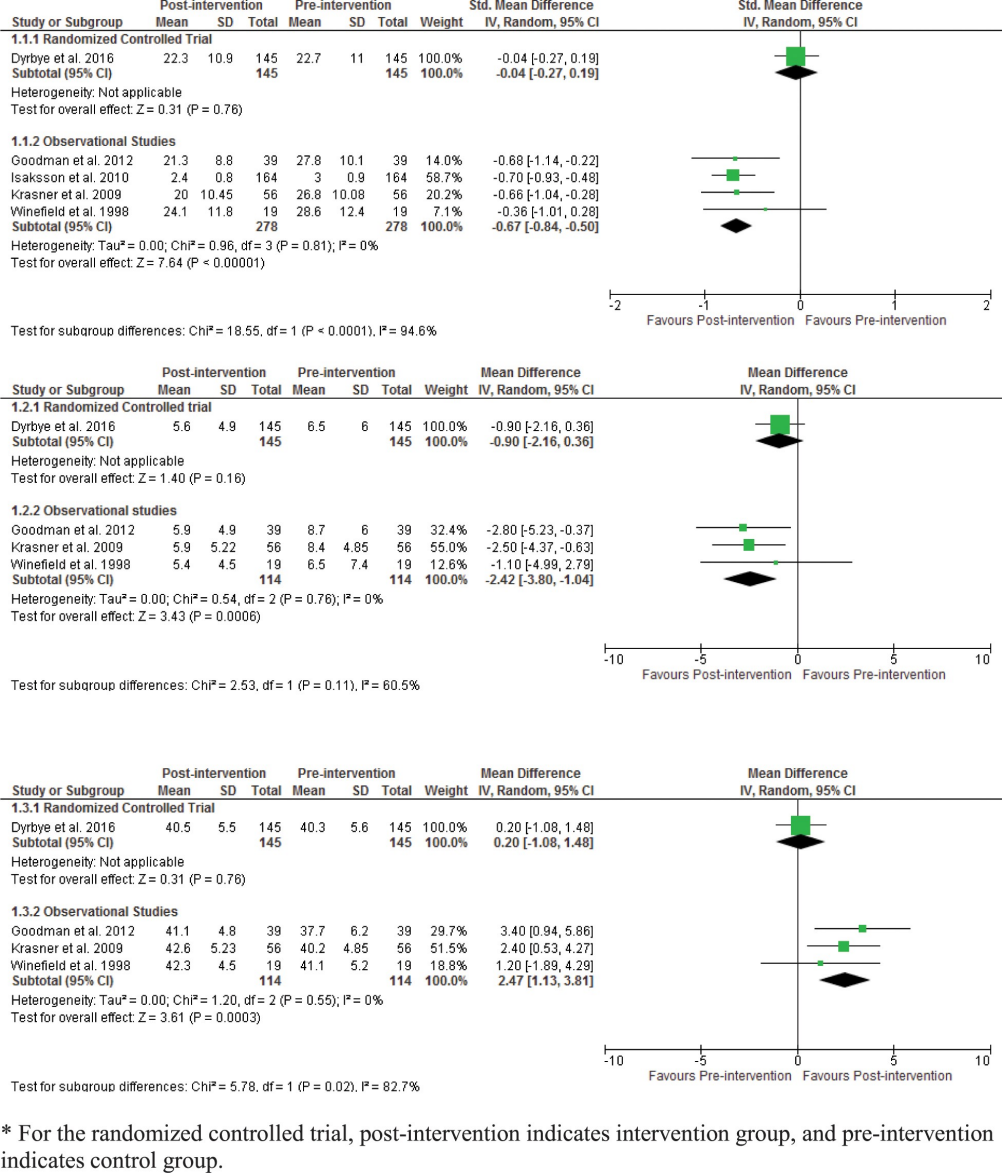
a. Pooled between-group differences in emotional exhaustion scores (burnout). b. Pooled between-group differences in depersonalization scores (burnout). c. Pooled between-group differences in personal accomplishment scores (burnout).

We were unable to conduct meta-analyses due to significant methodological heterogeneity in study designs, and inconsistency in the outcomes measured across studies. Only the results for burnout could be meta-analyzed. We present a forest plot for resilience measured by validated resilience scales but without an overall pooled effect measure due to heterogeneity (S1 File). We present a general forest plot for burnout for visual simplicity in Fig 2 including observational studies and one RCT. The RCT showed no statistically significant differences for all three burnout subscales. For the observational studies, 4 studies contributed to random effects meta-analysis for emotional exhaustion [pooled SMD -0.67 (95% CI -0.84 to -0.5) p = 0.81; I^2^ = 0%]. For the depersonalization subscale, 3 studies contributed to meta-analysis [pooled MD -2.42 (95% CI -3.80 to -1.04) p = 0.76; I^2^ = 0%]. For the personal accomplishment subscale, the same 3 studies contributed to meta-analysis [pooled MD 2.47 (95% CI 1.13 to 3.81) p = 0.55; I^2^ = 0%]. For these observational studies, all burnout subscales showed a statistically significant improvement.

Due to the small number of studies we were only able to conduct subgroup analysis for primary care physicians for burnout including only two studies [38,40] but results did not differ for any of the burnout subscales [EE: pooled SMD -0.58 (95% CI -0.91 to -0.25) p = 0.44; I^2^ = 0%. DP: pooled MD -2.24 (95% CI -3.92 to -0.56) p = 0.53; I^2^ = 0%. PA: pooled MD 2.08 (95% CI 0.48 to 3.68) p = 0.52; I^2^ = 0%] (S2 File).

### Assessment of publication bias and quality of evidence

We did not conduct funnel plots due to insufficient number of studies. While we undertook a broad search publication bias may be possible as we only included studies in English, French and Spanish. Most of the studies were not prone to selective reporting bias, however, a few of the studies were unclear. Most studies were funded with potential conflict of interests in two studies [41,42].

The quality of evidence for our outcome measures was assessed using GRADE. For resilience and MBI subscales (EE, DP and PA) it was estimated to be very low for RCT and low for observational studies (S5 Table). For the subsequent outcomes, two studies measured empathy. One was an observational study for which the quality was very low, but for the RCT, it was deemed low. Anxiety was only measured in one RCT and the quality was estimated as low. Depression was measured in 2 RCTs and it was deemed of moderate quality (S6 Table). The risk of bias for all outcomes measures was serious across studies. Most observational studies lacked a control group and confounding adjustment. Some studies had substantial loss to follow-up and for all studies blinding of participants and of the outcome assessment was not possible.

The risk of bias was similar between RCT studies (S3 Table). Three [37,43,44] out of 4 RCTs used computer-generated algorithms for randomization, but only one [44] had allocation concealment. Blinding of participants, study personnel and outcome assessment was deemed not possible for the four studies due to the nature of the intervention. Attrition bias was generally low. Reporting bias ranged from low to unclear. The within-study risk of bias for the 5 observational studies was also relatively similar between studies (S4 Table). Bias due to potential confounding was deemed serious for all 5 studies. Selection bias was deemed moderate in most observational studies [38–41], as selection into the study may have been related to the intervention and outcome, where physicians in greater need of the intervention may be more likely to participate. One [39] was a paid course, impacting selection of participants into the study. One study [41] had 19% lost to follow-up and reported that those participants had higher levels of distress (emotional exhaustion) and higher levels of emotion-focused coping strategies at baseline. Another study [40] had 20% loss to follow-up at the end of the study, and no information was given regarding characteristics or reasons for being lost to follow-up. Bias in measurement of outcomes was deemed moderate for all studies, as both blinding of participants and of the outcome assessment was not possible. Bias in selection of the reported results was generally low, but two studies [38,42] had serious bias due to incomplete presentation of results or imprecise p-values.

## Discussion

Overall, our systematic review provides insights into resilience interventions for physicians who have completed training. Physicians within training programs practice differently (e.g., protected learning time, working hours’ regulations, supervision) than physicians who have completed training who face unique challenges and pressures related to the responsibilities of independent practice. As such, we believe that the interventions applicable to building resilience in this group have different outcomes and merit separate study.

Specifically, this review demonstrates there is currently weak evidence to support one intervention over another to improve resilience. Interventions varied greatly in approach, duration, intensity and follow-up. Only two studies measured resilience using validated resilience scales, both of which provided definitions of resilience that matched our criteria. Both were small pilot studies with analyzable data specifically for physicians. Both studies reported statistically significant improvement in resilience scores, but they were of small sample sizes with one reporting substantial loss to follow-up. We cannot conclude about clinical significance with only two small studies in different physician specialties. We were unable to provide a pool estimate for resilience measured by validated resilience scales due to considerable clinical and statistical heterogeneity (I^2^ = 79%). Overall, we are uncertain of the effectiveness of these interventions to improve resilience.

For our secondary outcome measures, we found modest improvement in burnout. However, most studies were uncontrolled before-and-after studies with no confounding adjustment and relatively small sample sizes. The RCT that provided analyzable data for burnout had no significant improvements in burnout (all subscales). Thus, the slight improvement observed in the observational studies, was not enough to support any specific intervention towards improving burnout to enhance resilience. For other secondary outcome measures (empathy, depression and anxiety) data was lacking to conduct a meta-analysis. Only a few studies contributed to these outcomes and their samples sizes were small, limiting our ability to conclude with confidence the effect of the implemented interventions.

We conducted this systematic review in accordance with our registered protocol following PRISMA guidelines. Some studies did not present data in a way that could be extracted for meta-analysis, limiting our sample size and overall conclusions, and some studies included physicians from various levels of care limiting our ability to compare the effect of the intervention across these. Overall, there was such heterogeneity in various aspects of research design that drawing general recommendations was not possible. Furthermore, most of the observational studies that contributed data for meta-analysis did not have a control group, and many studies did not provide reasons and characteristics of those lost to follow-up. Overall, the quality of the evidence for all outcome measures was very low or low and risk of bias was serious, further limiting the strength of the evidence.

We only included English, French and Spanish articles, and all were small in sample size. Determining whether a study implemented an intervention to improve resilience by means of our secondary outcome measures was dependent on our interpretation, and authors’ presentation of their interventions and related outcome measures. Specifically, regarding burnout, we had to distinguish between studies that aimed to reduce trauma-induced stress from studies that reduced burnout to ultimately enhance resilience. Additionally, the small number of studies and high statistical heterogeneity limited our data analysis. However, as resilience interventions continue to be implemented in the health professions, this systematic review provides insights into ways that future studies can be designed or defined to ensure that we are able to identify effective resilience interventions.

## Conclusions

Resilience interventions have the potential to improve resilience among physicians, yet there is limited information available as to their efficacy and impact across studies. To our knowledge, this is the first systematic review to assess interventions to improve resilience specifically in physicians who have completed training, working in any setting. Our findings for burnout from observational studies showed a modest improvement in burnout, which is consistent with a recent systematic review [45] of interventions to prevent and reduce physician burnout. However, we did not find the same results for RCTs. We recommend that future research include large pragmatic RCTs with sufficient follow-up time. In addition, trials should define what they mean by resilience and whether the outcome measure actually measures resilience or another proxy outcome.

## Supporting information

S1 Checklist Research checklist–PRISMA guidelines. (DOCX)

S1 Database Database search strategies. (DOCX)

S1 Table Summary of methodological characteristics of studies including results specifically for physicians. (DOCX)

S2 Table Summary of studies that met inclusion criteria but their data could not be extracted for physicians. (DOCX)

S3 Table Within-study risk of bias in randomized controlled trials. (DOCX)

S4 Table Within-study risk of bias in observational studies. (DOCX)

S5 Table GRADE: Quality of evidence assessment for resilience and burnout subscales (emotional exhaustion, depersonalization and personal accomplishment). (DOCX)

S6 Table GRADE: Quality of evidence assessment for anxiety and depression subscales (emotional exhaustion, depersonalization and personal accomplishment). (DOCX)

S1 File Forest plot of mean differences for resilience scores–no pooling. (DOCX)

S2 File Subgroup analysis primary care physicians. (DOCX)

S3 File Sensitivity analysis for gender. (DOCX)

S4 File Subgroup analysis primary care burnout. (RM5)

S5 File Fig 2 data and analysis. (RM5)
