# Correction: Interventions to improve resilience in physicians who have completed training: A systematic review

**DOI:** 10.1371/journal.pone.0214782

**Published:** 2019-03-28

**Authors:** Carolina Lavin Venegas, Miriam N. Nkangu, Melissa C. Duffy, Dean A. Fergusson, Edward G. Spilg

The first author’s name appears incorrectly in the citation. The correct citation is: Lavin Venegas C, Nkangu MN, Duffy MC, Fergusson DA, Spilg EG (2019) Interventions to improve resilience in physicians who have completed training: A systematic review. PLoS ONE 14(1): e0210512. https://doi.org/10.1371/journal.pone.0210512
