# Supplementary material for: Interventions to improve resilience in physicians who have completed training: A systematic review
Source: PLoS One. 2019 Jan 17;14(1):e0210512. doi: 10.1371/journal.pone.0210512 (PMC6336384; doi:10.1371/journal.pone.0210512)
Supplement: S1 Database — (DOCX) [file pone.0210512.s002.docx]

**S1 Database. Database search strategies**Database: Ovid **MEDLINE**(R) In-Process & Other Non-Indexed Citations and Ovid MEDLINE(R) <1946 to Present>
Search Strategy:
--------------------------------------------------------------------------------
1    Resilience, Psychological/ (2952)
2    Adaptation, Psychological/ (83746)
3    resilien*.tw. (17555)
4    adaptation*.tw. (150267)
5    (coping adj3 behavio*).tw. (2674)
6    hardiness*.tw. (1125)
7   exp Social Adjustment/ (22357)
8    exp Physicians/ (112109)
9    physician*.tw. (323318)
10    1 or 2 or 3 or 4 or 5 or 6 or 7 (261465)
11    8 or 9 (389878)
12    10 and 11 (3956)

Database: **PsychINFO**<1806 to February Week 4 2017>
Search Strategy:
--------------------------------------------------------------------------------
1    "resilience (psychological)"/ or exp coping behavior/ (51262)
2    resilien*.ti,ab. (21500)
3    (cope or copes or coping).ti,ab. (80622)
4    1 or 2 or 3 (104962)
5    physicians/ or family physicians/ or general practitioners/ or gynecologists/ or internists/ or neurologists/ or obstetricians/ or pathologists/ or pediatricians/ or psychiatrists/ or surgeons/ or clinicians/ (47728)
6    physician*.tw. (57501)
7    5 or 6 (86185)
8    4 and 7 (2141)

Database: **EMBASE**<1974 to 2017 March 03>
Search Strategy:
--------------------------------------------------------------------------------
1    *physician/ (77032)
2    physician*.tw. (443692)
3    doctor*.tw. (145276)
4    or/1-3 (588007)
5    *coping behavior/ (16187)
6    resilien*.ti,ab. (20751)
7    (coping adj2 behav*).ti,ab. (2864)
8    or/5-7 (36604)
9    4 and 8 (1187)

Database: EBM Reviews - **Cochrane Database of Systematic Reviews** <2005 to March 1, 2017>, EBM Reviews - ACP Journal Club <1991 to February 2017>, EBM Reviews - Database of Abstracts of Reviews of Effects <1st Quarter 2016>, EBM Reviews - Cochrane Central Register of Controlled Trials <February 2017>, EBM Reviews - Cochrane Methodology Register <3rd Quarter 2012>, EBM Reviews - Health Technology Assessment <4th Quarter 2016>, EBM Reviews - NHS Economic Evaluation Database <1st Quarter 2016>
Search Strategy:
--------------------------------------------------------------------------------
1    Resilience, Psychological.mp. [mp=ti, ab, tx, kw, ct, ot, sh, hw] (126)
2    Adaptation, Psychological.mp. [mp=ti, ab, tx, kw, ct, ot, sh, hw] (3867)
3    resilien*.tw. (743)
4    adaptation*.tw. (6201)
5    (coping adj3 behavio*).tw. (482)
6    hardiness*.tw. (18)
7    exp Social Adjustment/ (883)
8    1 or 2 or 3 or 4 or 5 or 6 or 7 (11273)
9    exp Physicians/ (1518)
10    11 physician*.tw. (26)
11    9 or 10 (1542)
12    8 and 11 (12)

Database: **CINAHL**
Search Strategy:

--------------------------------------------------------------------------------

1    MH “Physicians”
2    AB physician*
3    AB doctor*
4    1 or 2 or 3
5    MH “hardiness”
6   AB resilien*
7    AB (coping N2 behav*)
8    AB hardiness*
9    5 or 6 or 7 or 8
10   4 and 9 (189)
