# Supplementary material for: Interventions to improve resilience in physicians who have completed training: A systematic review
Source: PLoS One. 2019 Jan 17;14(1):e0210512. doi: 10.1371/journal.pone.0210512 (PMC6336384; doi:10.1371/journal.pone.0210512)
Supplement: S1 Table — (DOCX) [file pone.0210512.s003.docx]

**S1 Table. Summary of methodological characteristics of studies including results specifically for physicians**

| Randomized controlled trials | | | | | | |
| --- | --- | --- | --- | --- | --- | --- |
| **Author/Year** | **Study Design** | **Randomized/ analyzed** | **Measurement timepoints and follow-up** | **Relevant outcomes** | **Relevant outcomes included in meta-analysis** | **Funding Sources** |
| Dyrbye et al. 2016 | RCT | 290/290  IG 145  CG 145 | Baseline and after 3 months | Burnout, depression | Burnout | Funded |
| Mache et al. 2016 | Pilot RCT | 76/72  IG 37  CG 35 | Baseline, 3 months and 6 months | Resilience | Not applicable | Not specified |
| Sood et al. 2011 | Pilot RCT | 40/32  IG 20  CG 12 | Baseline and after 8 weeks | Resilience, anxiety | Not applicable | Funded |
| West et al. 2014 | RCT | 74/72  IG 35  CG 37 | Baseline, every 3 months through the 9-month study intervention, and at 3 and 12 months following the study | Depression, empathy, burnout | Not applicable | Funded |
| Observational Studies | | | | | | |
| **Author/Year/** | **Study design** | **Sample size** | **Measurement timepoints and follow-up** | **Relevant outcomes** | **Relevant outcomes included in meta-analysis** | **Funding Sources** |
| Goodman et al. 2012 | Uncontrolled before-and-after | 93 health care providers  39 practicing physicians | Baseline and after 8 weeks | Burnout | Burnout | Not funded. Participants paid for the course |
| Krasner et al. 2009 | Uncontrolled before-and-after | Invited 871 Enrolled 70 Participated in last follow-up 56 | Baseline, pre-intervention, 8 weeks, 12 months and 15 months | Burnout, empathy | Burnout | Funded |
| Isaksson et al. 2010 | Prospective cohort | Eligible 242  Consented 227  Completed 184 | Baseline, 1 year and 3 years | Emotional exhaustion (burnout) | Burnout (EE only) | Funded |
| Sherlock et al. 2016 | Controlled before-and- after | Invited 314  Responded 225  Met baseline criteria 185 | Baseline, start of training, 3 months and 6 months | Anxiety, depression | Not applicable | Research grant |
| Winefield et al. 1998 | Uncontrolled before-and-after | Recruited 20  Analyzed 19 | Baseline and 4 weeks | Burnout | Burnout | Funded |

Abbreviations: IG, intervention group; CG, control group; EE, emotional exhaustion (burnout).
