# Supplementary material for: Interventions to improve resilience in physicians who have completed training: A systematic review
Source: PLoS One. 2019 Jan 17;14(1):e0210512. doi: 10.1371/journal.pone.0210512 (PMC6336384; doi:10.1371/journal.pone.0210512)
Supplement: S2 Table — (DOCX) [file pone.0210512.s004.docx]

**S2 Table. Summary of studies that met inclusion criteria but their data could not be extracted for physicians.**

| **Author** | **Year** | **Study Design** | **Population** | **Setting/Country** | **Intervention** | **Relevant outcome measures** | **Funding** | **Reason for exclusion from data analysis** |
| --- | --- | --- | --- | --- | --- | --- | --- | --- |
| Asuero et al. | 2013* | Uncontrolled before-and-after | Physicians, nurses, psychologists and social workers | Primary care professionals from Catalonia, Spain | Mindfulness program | Burnout and empathy | Partially funded | All studies met PICO criteria, but did not report subgroup analysis for physicians and thus, data was not possible to extract for analysis |
| Asuero et al. | 2014 | Pragmatic RCT | Nurses, physicians and other (practicing primary health care professionals) | Primary health care centers in Spain. | Mindfulness-based coping strategies, mindfulness practice, yoga, and  group discussions | Burnout and empathy | Not specified |  |
| Fortney et al. | 2013 | Uncontrolled before-and-after pilot study | Nurse practitioners, physician assistants and physicians (primary care clinicians) | University of Wisconsin -Madison departments of family medicine, internal  medicine, and pediatrics, USA. | Abbreviated mindfulness course | Resilience, burnout, depression and anxiety | Funded |  |
| Kemper et al. | 2015 | Prospective cohort | Dietitian, nurse, physician, physician assistant, social work, researcher, and others | Midwestern  academic health center, USA | Online mind–body skills training for resilience | Resilience, burnout and empathy | Funded |  |
| Mehta et al. | 2016 | Uncontrolled before-and-after pilot study | Physicians, nurse practitioners, nurses, and social workers | Massachusetts General Hospital, USA. | Relaxation Response Resiliency Program (3RP)- a mind-body program designed to promote resiliency | Resilience | Not specified |  |
| Schneider et al. | 2014 | Qualitative | Primary care and specialty-trained physicians, and a wide spectrum of professional stages from first-year residents to physicians in latter stages of their professional careers | Duke University Health System, USA | Physician well-being coaching adapted from Duke Integrative Medicine’s model | Resilience via skill and awareness development in the following three main areas: (1) boundary setting and prioritization, (2) self-compassion and self-care, and (3) self- awareness | Not specified |  |
| Shaphiro et al. | 2005 | Pilot RCT | Physicians, nurses, social workers,  physical therapists, and psychologists | Health care professionals  from the Palo Alto and Menlo Park  Divisions of the Veterans Affairs Palo Alto Health Care System, USA | Short-term stress  management program, mindfulness-based stress reduction (MBSR) | Burnout | Not specified |  |
| Sood et al. | 2014 | Pilot RCT | Physicians or scientists | Department of Radiology at the Mayo Clinic, Rochester, USA | Stress Management and Resiliency Training (SMART) program | Resilience | Not specified |  |

* Study in Spanish
