# Supplementary material for: Interventions to improve resilience in physicians who have completed training: A systematic review
Source: PLoS One. 2019 Jan 17;14(1):e0210512. doi: 10.1371/journal.pone.0210512 (PMC6336384; doi:10.1371/journal.pone.0210512)
Supplement: S3 Table — (DOCX) [file pone.0210512.s005.docx]

**S3 Table. Within-study risk of bias in randomized controlled trials.**

| **Study** | **Mache et al. 2016** | **Sood et al. 2011** | **West et al. 2014** | **Dyrbye et al. 2016** |
| --- | --- | --- | --- | --- |
| Selection bias -  Random sequence generation | Low - Randomization through a computer-generated algorithm | Unclear - Not described in sufficient detail | Low - Randomization through a computer-generated algorithm | Low - Randomization through a computer-generated algorithm |
| Selection bias-  Allocation concealment | Unclear - Not described in sufficient detail | Unclear - Not described in sufficient detail | Low – Concealed procedure | Unclear - Not described in sufficient detail |
| Performance bias -  Blinding (participants and personnel) | High^*^ - Blinding of participants and personnel was not possible | High^*^ - Blinding of participants and personnel was not possible | High^*^ - Blinding of participants and personnel was not possible | High^*^ - Blinding of participants and personnel was not possible |
| Detection bias -  Blinding (outcome assessment) | High^*^ - Self-reported outcomes | High^*^ - Self-reported outcomes | High^*^ - Self-reported outcomes | High^*^ - Self-reported outcomes |
| Attrition bias -  Incomplete outcome data | Low - Clearly stated number of excluded participants in each group and reasons for exclusion. | Low - Clearly stated number of participants randomized, number excluded in each group and reasons for exclusion | Low - Clear participant study flow reported. | Unclear - Clearly stated missing outcome data for all outcomes in each group, but did not provide reasons or characteristics |
| Reporting bias -  Selective reporting | Unclear - Unclear outcome reporting | Low - Selective outcome reporting bias not detected | Unclear - Unclear outcome reporting | Low - Selective outcome reporting bias not detected |
| Other bias -  Other sources of bias | Low- no other bias detected | Low- no other bias detected | Low- no other bias detected | Low- no other bias detected |

Using Cochrane Risk of Bias Tool
* Due to the nature of the intervention, blinding of study participants did not seem feasible and the outcomes were most likely required to be self-reported measures.
