# Supplementary material for: Interventions to improve resilience in physicians who have completed training: A systematic review
Source: PLoS One. 2019 Jan 17;14(1):e0210512. doi: 10.1371/journal.pone.0210512 (PMC6336384; doi:10.1371/journal.pone.0210512)
Supplement: S4 Table — (DOCX) [file pone.0210512.s006.docx]

**S4 Table. Within-study risk of bias in observational studies**

| **Study** | **Goodman et al. 2012** | **Krasner et al. 2009** | **Isaksson et al. 2010** | **Sherlock et al. 2016** | **Winefield et al. 1998** |
| --- | --- | --- | --- | --- | --- |
| Bias due to confounding | Serious | Serious | Serious | Serious | Serious |
| Bias in selection of participants into the study | Moderate | Moderate | Moderate | Low | Moderate |
| Bias in measurement of interventions | Low | Low | Low | Moderate | Low |
| Bias due to departures from intended interventions | Low | Low | Low | Low | Low |
| Bias due to missing data | Low | Moderate | Moderate | Low | Low |
| Bias in measurement of outcomes | Moderate | Moderate | Moderate | Moderate | Moderate |
| Bias in selection of the reported results | Low | Low | Low | Serious | Serious |

* Using A Cochrane Risk of Bias Assessment Tool: for Non- Randomized Studies of Interventions (ACROBAT-NRSI).
