# Supplementary material for: Interventions to improve resilience in physicians who have completed training: A systematic review
Source: PLoS One. 2019 Jan 17;14(1):e0210512. doi: 10.1371/journal.pone.0210512 (PMC6336384; doi:10.1371/journal.pone.0210512)
Supplement: S5 Table — (DOCX) [file pone.0210512.s007.docx]

**S5 Table. GRADE: Quality of evidence assessment for resilience and burnout subscales (emotional exhaustion, depersonalization and personal accomplishment).**

| **Quality assessment** | | | | | | | **№ of patients** | | **Effect** | **Quality** | **Importance** |
| --- | --- | --- | --- | --- | --- | --- | --- | --- | --- | --- | --- |
| **№ of studies** | **Study design** | **Risk of bias** | **Inconsistency** | **Indirectness** | **Imprecision** | **Other considerations** | **Physicians receive interventions** | **Nothing** | **Absolute (95% CI)** |  |  |
| Resilience (follow up: range 8 weeks to 6 months; assessed with: Brief Resilient Coping Scale and Connor Davidson Resilience Scale) | | | | | | | | | | | |
| 2 | Randomised trials  -Mache et al. 2016  -Sood et al. 2011 | serious ^a^ | very serious ^b^ | not serious | very serious ^c^ | none | 57 | 47 | Not pooled | ⨁◯◯◯VERY LOW | CRITICAL |
| Emotional Exhaustion (Burnout) (follow up: range 4 weeks to 15 months; assessed with: Maslach Burnout inventory) | | | | | | | | | | | |
| 4 | Observational studies  -Krasner et al. 2009 -Winefield et al. 1998 - Isaksson et al. 2010  - Goodman et al. 2012 | serious ^d,e^ | not serious | not serious | serious ^f^ | No adjustment for confounding | 278 | 278 | SMD **0.67 SD lower** (0.84 lower to 0.5 lower) | ⨁⨁◯◯LOW | IMPORTANT |
| Emotional Exhaustion (Burnout) (follow up: mean 6 months; assessed with: Maslach Burnout inventory) | | | | | | | | | | | |
| 1 | Randomised trials  - Dyrbye et al. 2016 | serious ^a,g^ | not serious | not serious | very serious ^f^ | none | 145 | 145 | SMD **0.04 SD lower** (0.27 lower to 0.19 higher) | ⨁◯◯◯ VERY LOW | IMPORTANT |
| Depersonalization (Burnout) (follow up: range 4 weeks to 15 months; assessed with: Maslach Burnout inventory) | | | | | | | | | | | |
| 3 | Observational studies  -Krasner et al. 2009 -Winefield et al. 1998 - Goodman et al. 2012 | serious ^h^ | not serious | not serious | serious ^f^ | No adjustment for confounding | 114 | 114 | MD **2.42 lower** (3.8 lower to 1.04 lower) | ⨁⨁◯◯ LOW | IMPORTANT |
| Depersonalization (Burnout) (follow up: mean 6 months; assessed with: Maslach Burnout inventory) | | | | | | | | | | | |
| 1 | Randomised trials  - Dyrbye et al. 2016 | serious ^a,g^ | not serious | not serious | very serious ^f^ | none | 145 | 145 | MD **0.9 lower** (2.16 lower to 0.36 higher) | ⨁◯◯◯ VERY LOW | IMPORTANT |
| Personal Accomplishment (Burnout) (follow up: range 4 weeks to 15 months; assessed with: Maslach Burnout inventory) | | | | | | | | | | | |
| 2 | Observational studies  -Krasner et al. 2009 -Winefield et al. 1998 - Goodman et al. 2012 | serious ^h^ | not serious | not serious | serious ^f^ | No adjustment for confounding | 114 | 114 | MD **2.47 higher** (1.13 higher to 3.81 higher) | ⨁⨁◯◯ LOW | IMPORTANT |
| Personal Accomplishment (Burnout) (follow up: mean 6 months; assessed with: Maslach Burnout inventory) | | | | | | | | | | | |
| 1 | Randomised trials  - Dyrbye et al. 2016 | serious ^c,g^ | not serious | not serious | very serious ^f^ | none | 145 | 145 | MD **0.2 higher** (1.08 lower to 1.48 higher) | ⨁◯◯◯ VERY LOW | IMPORTANT |

**CI:** Confidence interval; **SMD:** Standardised mean difference; **MD:** Mean difference

#### Explanations

a. No allocation concealment. No clear randomization process for one study. No blinding of participants or during outcome assessment.

b. I^2^=79%. Not much overlap in confidence intervals. Inconsistent effect estimates.

c. Pilot studies with few participants. Confidence interval for Mache et al. crosses the null.

d. Substantial loss to follow-up.

e. No adjustment for confounding and no control group.

f. Small sample size (<300 participants provide data for the outcome).

g. No explanation regarding characteristics of those with missing data or reasons for being lost to follow-up.

h. No adjustment for potential confounding. Krasner et al. did not provide enough information for missing data.
