# Supplementary material for: Interventions to improve resilience in physicians who have completed training: A systematic review
Source: PLoS One. 2019 Jan 17;14(1):e0210512. doi: 10.1371/journal.pone.0210512 (PMC6336384; doi:10.1371/journal.pone.0210512)
Supplement: S6 Table — (DOCX) [file pone.0210512.s008.docx]

**S6 Table. GRADE: Quality of evidence assessment for anxiety and depression subscales (emotional exhaustion, depersonalization and personal accomplishment).**

| **Quality assessment** | | | | | | | **№ of patients** | | **Effect** | **Quality** | **Importance** |
| --- | --- | --- | --- | --- | --- | --- | --- | --- | --- | --- | --- |
| **№ of studies** | **Study design** | **Risk of bias** | **Inconsistency** | **Indirectness** | **Imprecision** | **Other considerations** | **Inter-vention** | **nothing** | **Absolute (95% CI)** |  |  |
| Anxiety (follow up: mean 8 weeks; assessed with: Smith Anxiety Scale) | | | | | | | | | | | |
| 1 | Randomised trials  - Sood et al. 2011 | serious ^a^ | not serious | not serious | serious ^b^ | none | 20 | 12 | MD **10 lower** (24.46 lower to 4.46 higher) | ⨁⨁◯◯ LOW | IMPORTANT |
| Depression (follow up: range 3 months to 12 months; assessed with: 2-item Primary Care Evaluation of Mental Disorders and the 2-question approach described by Spitzer et al and validated by Whooley et al. respectively) | | | | | | | | | | | |
|  | Randomised trials  - West et al. 2014  - Dyrbye et al. 2016 | serious ^c^ | not serious | not serious | not serious | none | Dyrbye et al. reported 28.2% of participants in the control group screened positive for depression compared to 21.9% in the intervention group at the end of the study (3 months). They reported the difference was not statistically significant. West et al. reported percent change for positive depression screening 12-months post-intervention. The control group had a reduction of 4.1% and the intervention group of 6.2%, which was not statistically significant. | | | ⨁⨁⨁◯MODERATE | IMPORTANT |
| Empathy (follow up: mean 12 months; assessed with: Jefferson scale of physician) | | | | | | | | | | | |
| 1 | Randomised trials  - West et al. 2014 | serious | not serious | not serious | serious ^d^ | none | Data not reported. Authors reported that there were no statistically significant differences in empathy. | | | ⨁⨁◯◯ LOW | IMPORTANT |
| Empathy (follow up: mean 15 months; assessed with: Jefferson Scale of Physician) | | | | | | | | | | | |
| 1 | Observational studies  - Krasner et al. 2009 | serious ^e^ | not serious | not serious | very serious ^d^ | strong association all plausible residual confounding would suggest spurious effect, while no effect was observed | 56 | 56 | MD **4.6 higher** (1.21 higher to 7.99 higher) | ⨁◯◯◯VERY LOW | IMPORTANT |

**CI:** Confidence interval; **MD:** Mean difference

#### Explanations

a. Unclear randomization sequence generation and allocation concealment.

b. Pilot study. Very small sample size. Confidence interval crosses the null.

c. Unclear allocation concealment and reasons for missing data for Dyrbye et al.

d. Very small sample size

e. Substantial missing data. No adjustment for potential confounding.
