# Supplementary figures and images for: Interventions to improve resilience in physicians who have completed training: A systematic review
Source: PLoS One. 2019 Jan 17;14(1):e0210512. doi: 10.1371/journal.pone.0210512 (PMC6336384; doi:10.1371/journal.pone.0210512)

**S1 File. Forest plot of mean differences for resilience scores – no pooling**


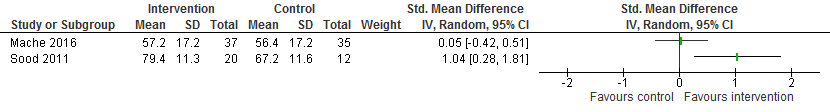

Supplement: S1 File — (DOCX) [file pone.0210512.s009.docx]
