# Supplementary material for: Interventions to improve resilience in physicians who have completed training: A systematic review
Source: PLoS One. 2019 Jan 17;14(1):e0210512. doi: 10.1371/journal.pone.0210512 (PMC6336384; doi:10.1371/journal.pone.0210512)

**S2 File. Subgroup analysis primary care physicians**

Subgroup analysis for emotional exhaustion (burnout) for primary care physicians.


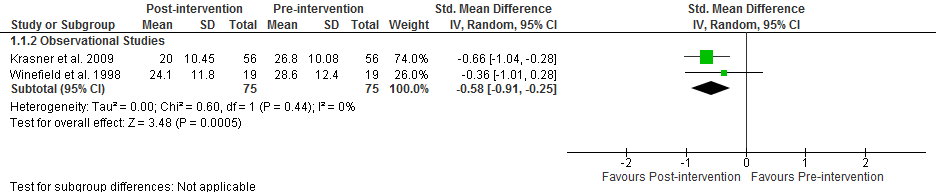


Subgroup analysis for depersonalization (burnout) for primary care physicians.


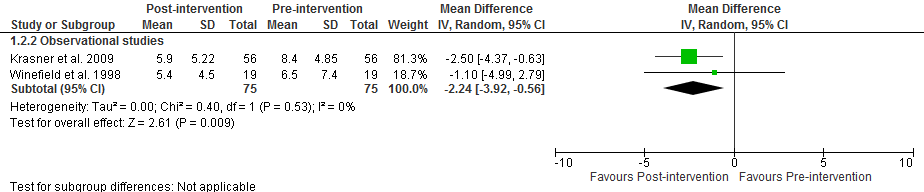


Subgroup analysis for personal accomplishment (burnout) for primary care physicians.


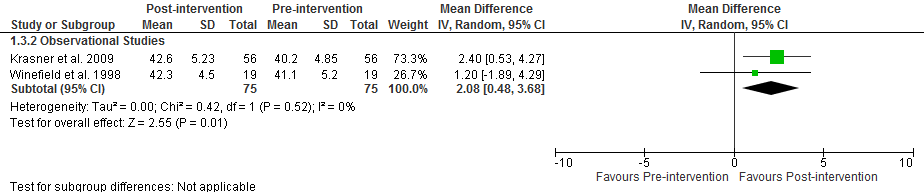

Supplement: S2 File — (DOCX) [file pone.0210512.s010.docx]
