# Supplementary material for: Interventions to improve resilience in physicians who have completed training: A systematic review
Source: PLoS One. 2019 Jan 17;14(1):e0210512. doi: 10.1371/journal.pone.0210512 (PMC6336384; doi:10.1371/journal.pone.0210512)

**S3 Sensitivity analysis for gender**

Sensitivity analysis for emotional exhaustion (burnout) excluding study done only in females.


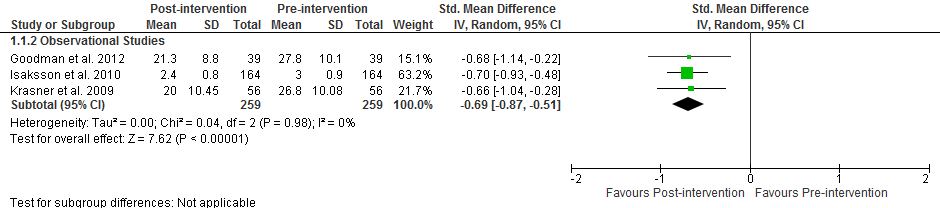


Sensitivity analysis for depersonalization (burnout) excluding study done only in females.


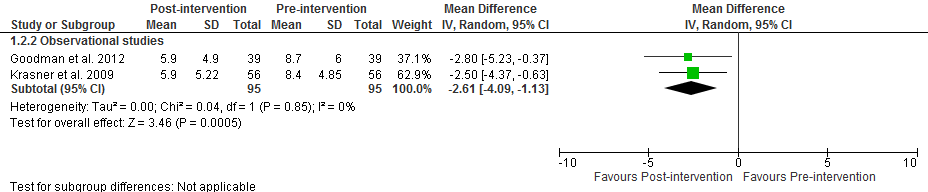


Sensitivity analysis for personal accomplishment (burnout) excluding study done only in females.


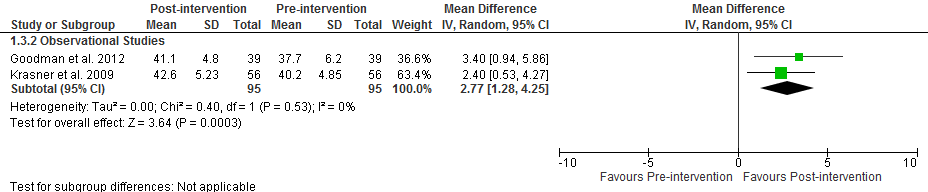

Supplement: S3 File — (DOCX) [file pone.0210512.s011.docx]
